# [Corrigendum] miR‑1299/NOTCH3/TUG1 feedback loop contributes to the malignant proliferation of ovarian cancer

**DOI:** 10.3892/or.2023.8533

**Published:** 2023-03-23

**Authors:** Yuqing Pei, Kexin Li, Xiaoying Lou, Yue Wu, Xin Dong, Wenpeng Wang, Ning Li, Donghong Zhang, Wei Cui

Oncol Rep 44: 438–448, 2020; DOI: 10.3892/or.2020.7623

Following the publication of the above article, a concerned reader drew to the authors' attention that the data shown for the ‘CAOV3/NC mimics’ experiment in Fig. 2D on p. 443 appeared to be the same as that shown for the ‘TUG1-sh+miR-1299 inhibitors’ experiment in Fig. 4H on p. 444.

The authors have examined their original data, and realize that the same data was inadvertently included in the two figures. Consequently, the corrected version of Fig. 2, featuring the correct data for the ‘CAOV3/NC mimics’ experiment in Fig. 2D, is shown opposite. The overall conclusions of this study were not affected by this error. All the authors agree to the publication of this corrigendum, and are grateful to the Editor of *Oncology Reports* for allowing them the opportunity to publish this; furthermore, they apologize to the readership for any inconvenience caused.

## Figures and Tables

**Figure 2.**
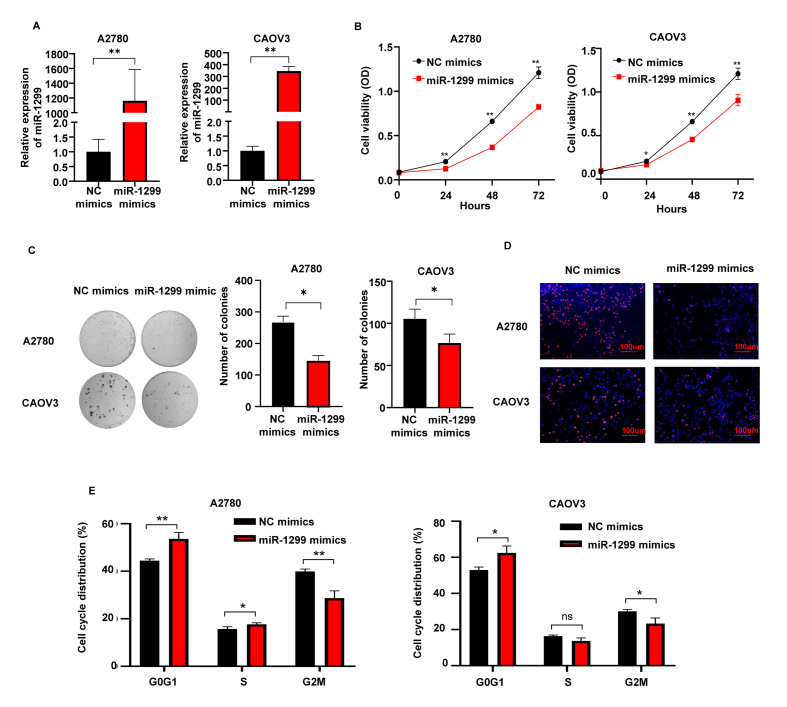
Upregulation of miR-1299 inhibits cell proliferation, colony formation, EdU incorporation, and cell cycle in OC A2780 and CAOV3 cell lines. (A) RT-qPCR analysis of miR-1299 expression level in OC cells after transfection of NC or miR-1299 mimics for 48 h. (B) Cell proliferation of OC cells after transfection of NC or miR-1299 mimics by CCK-8 assay. (C) Representative photographs and quantifications of the colony formation assay after transfection of NC or miR-1299 mimics. (D) Representative photographs of EdU incorporation assay in OC cells after transfection of NC or miR-1299 mimics. (E) Cell cycle distribution of OC cells after transfection of NC or miR-1299 mimics by flow cytometry. Means ± SD are shown. Statistical analysis was conducted using Student's t-test. *P<0.05 and **P<0.01, miR-1299 mimic compared with the NC mimic; ns, not significant; OC, ovarian cancer.

